# Isolation and sequencing of swine carbonic anhydrase VI, an enzyme expressed in the swine kidney

**DOI:** 10.1186/1756-0500-7-116

**Published:** 2014-02-28

**Authors:** Toshiho Nishita, Juro Yatsu, Masaru Murakami, Shino Kamoshida, Kensuke Orito, Nobutune Ichihara, Kazuyoshi Arishima, Hideharu Ochiai

**Affiliations:** 1 Laboratory of Physiology I, School of Veterinary Medicine, Azabu University, 1-17-71 Fuchinobe, Sagamihara, Kanagawa 252-5201, Japan; 2 Miyagi Prefectural Meat Sanitation Inspection Station, 314 Imaizumi, Sakuraoka, Yoneyamacho, Tome-city, Miyagi 987-0031, Japan; 3 Laboratory of Molecular Biology, School of Veterinary Medicine, Azabu University, 1-17-71 Fuchinobe, Sagamihara, Kanagawa 252-5201, Japan; 4 Laboratory of Physiology II, School of Veterinary Medicine, Azabu University, 1-17-71 Fuchinobe, Sagamihara, Kanagawa 252-5201, Japan; 5 Laboratory of Anatomy I, School of Veterinary Medicine, Azabu University, 1-17-71 Fuchinobe, Sagamihara, Kanagawa 252-5201, Japan; 6 Laboratory of Anatomy II, School of Veterinary Medicine, Azabu University, 1-17-71 Fuchinobe, Sagamihara, Kanagawa 252-5201, Japan; 7 Research Institute of Biosciences, School of Veterinary Medicine, Azabu University, 1-17-71 Fuchinobe, Sagamihara, Kanagawa 252-5201, Japan

**Keywords:** Carbonic anhydrase VI, cDNA, Swine kidney, mRNA, RT-PCR, Kidney disease

## Abstract

**Background:**

Carbonic anhydrase VI (CA-VI) is produced by the salivary gland and is secreted into the saliva. Although CA-VI is found in the epithelial cells of distal straight tubule of swine kidneys, the exact function of CA-VI in the kidneys remains unclear.

**Results:**

CA-VI was located in the epithelial cells of distal straight tubule of swine kidneys.

A full-length cDNA clone of CA-VI was generated from the swine parotid gland by reverse transcription polymerase chain reaction, using degenerate primers designed based on conserved regions of the same locus in human and bovine tissues.

The cDNA sequence was 1348 base pairs long and was predicted to encode a 317 amino acid polypeptide with a putative signal peptide of 17 amino acids. The deduced amino acid sequence of mature CA-VI was most similar (77.4%) to that of human CA-VI. CA-VI expression was confirmed in both normal and nephritic kidneys, as well as parotid. As the primers used in this study spanned two exons, the influence of genomic DNA was not detected. The expression of CA-VI was demonstrated in both normal and nephritic kidneys, and mRNA of CA-VI in the normal kidneys which was the normalised to an endogenous *β*–actin was 0.098 ± 0.047, while it was significantly lower in the diseased kidneys (0.012 ± 0.007). The level of CA-VI mRNA in normal kidneys was 19-fold lower than that of the parotid gland (1.887).

**Conclusions:**

The localisation of CA-VI indicates that it may play a specialised role in the kidney.

## Background

Carbonic anhydrase (CA; EC 4.2.1.1) is a well-characterised enzyme that catalyses the reversible hydration of CO_2_ to form HCO_3_^-^ and protons according to the following reaction: CO_2_ + H_2_O ↔ H_2_CO_3_ ↔ HCO_3_^-^ + H^+^. The first reaction is catalysed by CA and the second reaction occurs instantaneously. The mammalian α-CA gene family includes at least 15 enzymatically active isoforms with different structural and catalytic properties. Six of the active CA isozymes are cytosolic (CA-I, -II, -III, -VII, -VIII, and -XIII), 4 are membrane-associated (CA-IV, -IX, -XII, and -XIV), 2 are mitochondrial (CA-VA and CA-VB), and 1 is secretory form (CA-VI), while 2 CA-related proteins (CA-X and XI) are inactive variants [1-3]. The physiological function of carbonic anhydrase is to maintain the acid–base balance in various tissues and biological fluids [4].

CA-VI has been previously purified from the saliva and parotid glands of sheep [5] humans [6], cattle [7], pigs [8], and dogs [9]. The enzyme is localised in the serous acinar and demilune cells of the parotid and submandibular glands [7,10], from which it is secreted into saliva. CA-VI may participate in the regulation of salivary pH and buffer capacity, and protect the mouth and upper alimentary canal against excess acidity [11]. On the other hand, Hooper et al. [12] suggested that the unique oligosaccharide structures on bovine CA-VI might have an antibacterial function. Karhumaa et al. [13] also suggested that the glycoproteins on CA-VI confer multifunctionality on the enzyme.

To date, human, bovine, mouse, canine, and equine CA-VI cDNAs have been cloned successfully [14-18]. CA-VI was previously reported to be present in the parotid gland, saliva, bile, and serum of pigs [19]. However, the exact physiological and clinical significance of swine CA-VI has not been established. Here, we demonstrated immunohistochemical localization of CA-VI in the swine kidney and deduced the nucleotide sequence of swine CA-VI. Furthermore, we demonstrate expression of CA-VI in both normal and diseased kidneys of pigs. These data provide an initial step toward exploring the physiological and pathological roles of CA-VI.

## Methods

### Tissue samples

Samples from normal kidneys (n = 9), parotid gland (n = 1), and diseased kidneys (n = 19) of domestic pigs were taken from a slaughterhouse in Miyagi prefecture (Japan).

Macroscopically, diseased swine kidneys showed signs of necrosis (n = 7), nephritis (n = 7), pyelectasis (n = 3), and cystic kidney (n = 2).

All samples were obtained in accordance with the guidelines of the Laboratory Animal Care Committee of Azabu University, Japan, and programs accredited by the Office of Laboratory Animal Welfare (OLAW) USA (#A5393-01) were used.

The samples were immediately fixed in neutralised 10% formalin and Bouin’s solution, dehydrated with a graded series of alcohols, cleared with xylene, and then embedded in paraffin wax blocks that were cut into 4-μm-thick histological sections. To observe the morphologic changes, renal tissue samples were stained with hematoxylin and eosin. Microscopic inspection of diseased kidney revealed predominantly renal tubule necrosis and stromal cell permeation.

### Immunohistochemical staining of CA-VI in kidney

Biopsies of pig kidneys were performed. The samples were immediately fixed in neutralized 10% formalin and Bouin’s solution, dehydrated with a graded series of alcohols, cleared with xylene and then embedded in paraffin wax blocks that were cut into 4 μm-thick histological sections.

Endogenous peroxidase activity was blocked in deparaffinized and rehydrated sections using 0.3% H_2_O_2_ in methanol, and immersion in normal goat serum (2% in PBS) for 20 min blocked fragment crystallizable receptors. Monospecific primary antisera (diluted 1:2000) against swine CA-VI produced in our laboratory [8] was used to detect the respective isozymes during a 1-h incubation. Antibody binding was visualized using the Vectastain Elite avidin-biotin-peroxidase complex kit (ABC-POD reagent kit; Vector) and diaminobenzidine (DAB) according to the manufacturer’s protocol.

The kidney sections were stained with hematoxylin, dehydrated through a graded alcohol series, and mounted on coverslips.

Samples were observed and photographed under a light microscope.

### cDNA sequence of pig CA-VI

Total RNA was isolated from the parotid gland of a healthy pig by using RNA extraction solution (Isogen; Nippon Gene, Japan). Degenerate primers used for the amplification of a central region of swine CA-VI cDNA were designed based on the conserved sequences in human [14] and bovine [15] cDNAs. Reverse transcription- polymerase chain reaction (RT-PCR) amplification was then performed using the SuperScript One-Step RT-PCR system (Life Technologies, MS, USA), according to the manufacturer’s instructions. The RT-PCR products were verified to be single bands on agarose gel electrophoresis and were then purified using Ultrafree-DA (Millipore, MA, USA) and Microcon YM-100 (Millipore). The purified DNA was cloned into a pGEM-T Easy cloning vector (Promega, WI, USA) and sequenced using a Thermo Sequenase Fluorescent Labeled Primer Cycle sequencing kit (Amersham Biosciences, NJ, USA) and a DSQ2000L DNA sequencer (Shimadzu, Japan). In order to minimise PCR errors, sequences from several clones were analysed. The consensus nucleotide sequence showed a high similarity of approximately 80% to bovine CA-VI cDNA sequences. In order to amplify the 3′ and 5′ regions of pig CA-VI cDNA, 3′- and 5′ -rapid amplification of cDNA ends (3′- and 5′-RACE) methods were employed. 3′-RACE was performed as previously described [20] and 5′-RACE was carried out using a SMART RACE cDNA amplification kit (Clontech, CA, USA), according to the manufacturer’s protocol.

Each RACE product was cloned into the pGEM-T Easy vector and sequenced by employing an AmpliTaq Dye Terminator Cycle Sequencing FS Ready Reaction kit (Applied Biosystems, CA, USA) on a 373A DNA sequencer (Applied Biosystems).

### RNA extraction from FFPE tissue

A total of 19 diseased and 9 healthy kidney samples in formalin-fixed, paraffin-embedded (FFPE) were used in this study. The 4 pieces of 10-μm-thick FFPE sections were cut from each paraffin block and collected in a 1.5-mL tube. A NucleoSpin FFPE RNA isolation kit for FFPE Tissues (Takara, Kyoto, Japan) was then used according to the manufacturer’s protocol. Briefly, 1 mL of xylene was added into the 4 pieces of 20-μm-thick FFPE sections to remove traces of paraffin. The tissues were digested with proteinase K at 60°C for 3 h and treated with DNase I. After washing, total RNA, including a small miRNA fraction, was eluted with distilled water and stored at -80°C until use.

### cDNA synthesis and PCR evaluation of pig CA-VI

Reverse transcription was performed using a SuperScript III First-Strand Synthesis System (Invitrogen, Carlsbad, CA) according to the manufacturer protocol, and the resulting cDNA was used as a template for RT-PCR. Primer set used was purchased from Takara (Kyoto, Japan). Primer sequences for the CA-VI genes were following; forward: 5′-AGAATGTCCACTGGTTTGTGCTTG-3′; reverse: 5′-GGATGGTCTTGTTCTGGTCATTCA-3′. The expected product size of the PCR using this primer set is 102 bp. PCR reaction was performed using Takara *Ex Taq*™ Hot Start version (Takara, Kyoto, Japan). Amplification was conducted using the following protocol: initial denaturation phase at 95°C for 30 s, and then 40 cycles at 95°C for 15 s for denaturation, then at 60°C for 30 s for annealing and extension step. PCR products were loaded in 3% agarose gel.

### Real-time PCR evaluation of pig CA-VI

Real-time quantitative PCR was performed using a Thermal Cycler Dice® Real Time System II (Takara). Samples (final volume of 25 μL) were run in duplicate and contained the following: X1 SYBR® *Premix Ex Taq*™ II (Takara) 1 μL 10 mM of each primer and 2 μL cDNA template. Amplification conditions were carried out as manufacturer’s protocol. The primer set used in CA-VI amplification was the same as described above. The housekeeping gene β-actin was used as a reference gene (forward: 5′-TCTGGCACCACACCTTCT-3′, reverse; 5′-TGATCTGGGTCATCTTCTCAC-3′; DDBJ accession number AY550069). The Real-Time RT-PCR results are presented as the gene expression of the target gene (CA-VI) relative to that of the housekeeping gene (β-actin), and CA-VI gene expression levels are achieved using the 2^-ΔΔCT^ method of quantification [21].

### Statistical analysis

To compare differences in the relative levels of CA-VI mRNA between normal and nephritic kidneys, statistical analysis was carried out using an unpaired *t*-test. Values of P < 0.01 were considered to be statistically significant.

## Results

### Immunohistochemical study

**Figure 1:**
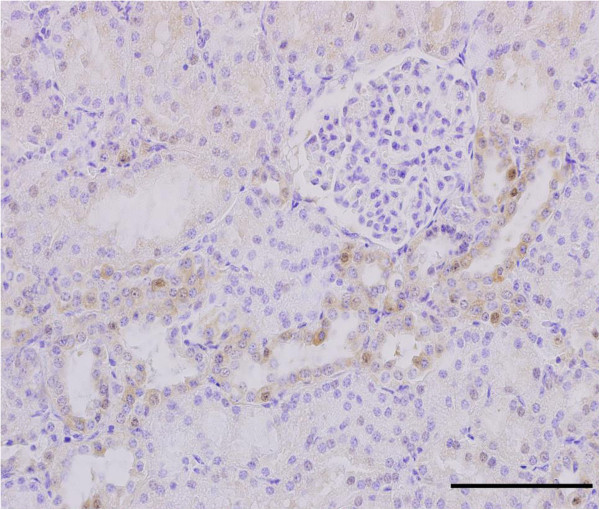
**Immunohistochemical localization of CA-VI in the swine kidney.** CA-VI was found in the epithelial cells of the distal straight tubule of swine kidneys. Scale bar: 50 μm.

The results of immunohistochemical localization of CA-VI in the kidneys of clinically normal pigs were shown in Figure 1. CA-VI was located in the epithelial cells of distal straight tubule of swine kidneys.

### Nucleotide sequence of swine CA-VI cDNA

**Figure 2:**
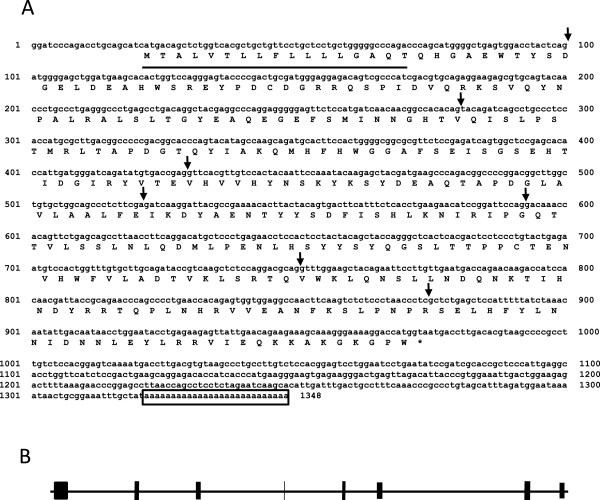
**Nucleotide and deduced amino acid sequences of swine *CA-VI *cDNA.** The DDBJ accession number is AB333806. The termination codon is asterisked and the polyadenylation signal is boxed. The putative signal peptides consisting of 17 amino acid is underlined. Arrows indicate the positions of introns **(A)**. Alignment of the cDNA sequence with swine genome chromosome 1 predicts 8 exons of 70–447 base pairs. Exon ■: intron ▬**(B)**.

A 1348-bp nucleotide sequence corresponding to full-length swine CA-VI cDNA was obtained (accession number AB333806), consisting of a 951-bp open reading frame encoding swine CA-VI of 317 amino acids (Figure 2A). A typical polyadenylation signal was found in the 3′ untranslated region. The deduced 317 amino acids included a signal peptide (17 amino acids) typical of most secreted proteins where the region was enriched with hydrophobic residues [22]; thus, the predicted mature protein consisted of 300 amino acids. To determine the genomic structure, the UCSC genome browser site (http://genome.ucsc.edu/) was used to align canine genomic sequences and the cDNA sequence of CA-VI (Figure 2B).

**Figure 3:**
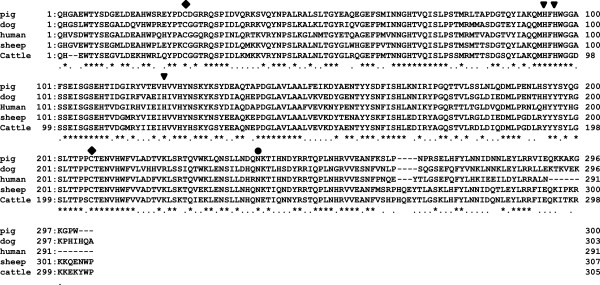
**Comparison of the amino acid sequences of mature CA-VI in pigs, dogs, humans, sheep, and cattle.** Multiple sequence alignments were performed using the Genetyx program (version 12). Asterisks and dots indicate identical residues and conservative substitution, respectively. Cys residues are indicated by diamonds, and His residues, by triangles. These are likely to form an intra-molecular disulphide bond and to bind to zinc, respectively. The potential glycosylation sites are indicated by circles.

The amino acid sequence of the deduced swine mature CA-VI was 3 residues shorter than that of canine CA-VI, and 9 residues (in the carboxy-terminal region) longer than that of mouse and human CA-VI, respectively. The sequence of swine CA-VI showed approximately 77.4% identity to human CA-VI (Figure 3). Two cysteine residues (amino acid positions 25 and 207), which are known to form intra-molecular disulphide bonds in sheep CA-VI [23], are conserved, and 3 histidine residues (amino acid positions 94, 96, and 121) responsible for zinc binding were also found. In addition, 2 potential N-glycosylation sites (Asn-X-Thr/Ser) were detected; 1 of these (amino acid positions 239–241, Asn-Lys-Thr) is known to be glycosylated in sheep CA-VI [23].

### Expression of swine CA-VI mRNA in kidney

**Figure 4:**
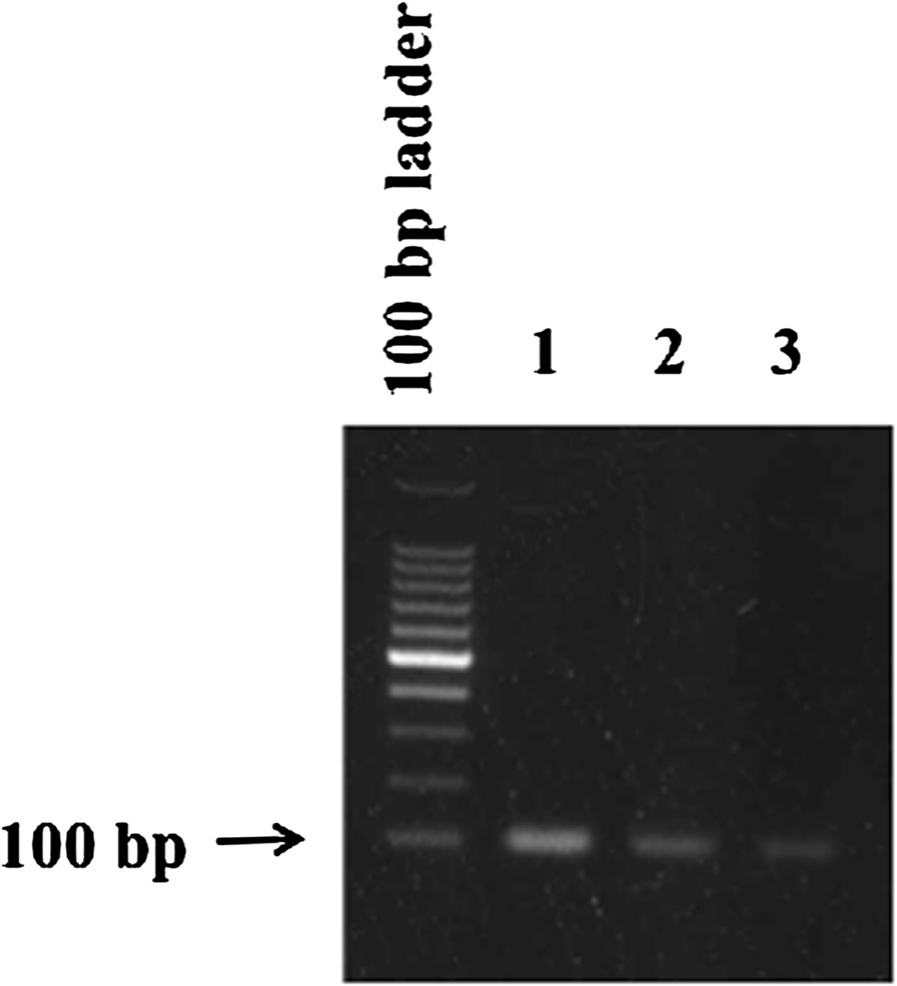
**RT-PCR analysis of CA-VI expression from FFPE samples.** m; 100-bp ladder, 1; parotid, 2; normal kidney, 3; nephritic kidney.

Figure 4 shows the RT-PCR analysis of CA-VI expression from FFPE samples. CA-VI expression was confirmed in both normal and nephritic kidneys, as well as parotid. As the primers used in this study spanned two exons, the influence of genomic DNA was not detected.

### The levels of CA-VI mRNA in the kidney

**Figure 5:**
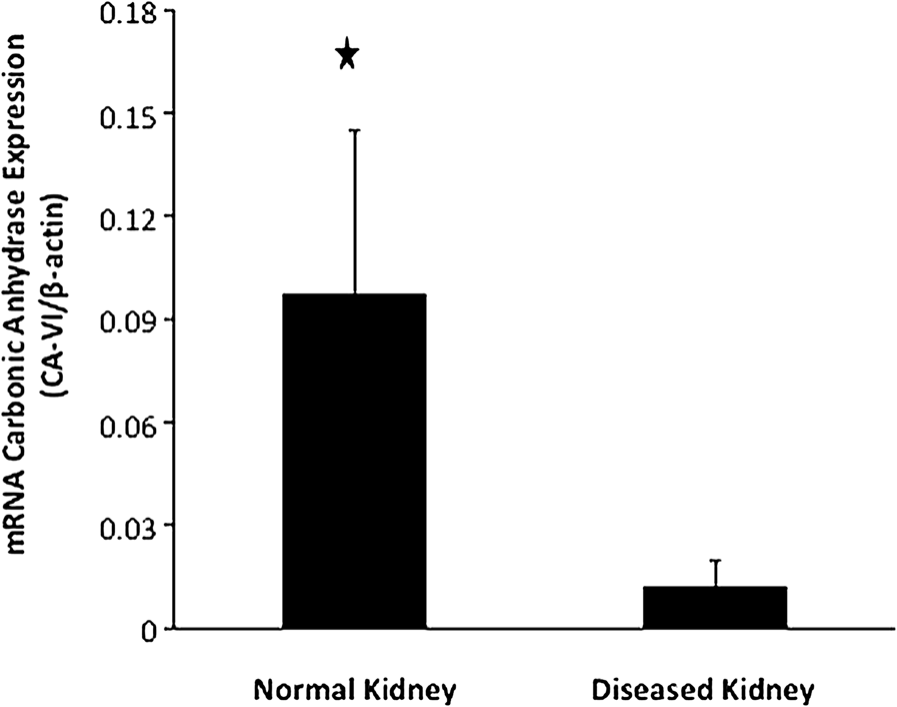
**Comparison of CA-VI expression in normal or diseased kidneys from FFPE samples as shown by quantitative RT-PCR.** CA-VI levels were normalised to an endogenous β-actin mRNA. The values represent the mean ± SD. Asterisks indicate a significant difference between the normal and diseased kidneys (*P* < 0.05, unpaired *t*-test).

Expression levels of CA-VI mRNA were measured by qRT-PCR in FFPE samples of normal and diseased kidneys (Figure 4). The relative level of CA-VI mRNA in the normal kidneys was 0.098 ± 0.047, while it was significantly lower in the diseased kidneys (0.012 ± 0.007; *p* = 2.71 × 10^-8^, Figure 5). The level of CA-VI mRNA in normal kidneys was 19-fold lower than that of the parotid gland (1.887).

## Discussion

Fernley et al., [24] reported that CA-VI was absent from the sublingual salivary gland, kidney, lung, adrenal, brain, skeletal muscle, liver, heart, pancreas, small intestine, and cerebrospinal fluid of sheep. However, CA-VI was found in the lung, skeletal muscle, liver, heart, and pancreas of pigs by using ELISA [19]. In the present study, we show for the first time that CA-VI is expressed in the epithelial cells of distal straight tubule of swine kidneys.

The expression of bovine CA-VI mRNA has previously been detected in the parotid gland, liver, and mammary gland of cow [25,26]. Canine CA-VI mRNA signals were strong in the major salivary glands and weaker in the minor salivary glands and esophagus, and were absent in the pancreas, liver, and almost all parts of the digestive tract, except the esophagus [17]. In the horse, CA-VI mRNA was detected in the digestive tract, salivary glands, testis, thyroid gland, and liver, but not in nerve tissue, skeletal muscle, spleen, or lymph node [18].

To our knowledge, there have been no previous studies on CA-VI mRNA expression in normal and diseased kidneys. Although the levels of CA-VI mRNA were lower in diseased kidneys, further studies are necessary to determine whether CA-VI is a suitable biomarker for kidney disorders.

In the kidney, cytosolic CA-II accounts for >95% of all CA activity. In humans, rabbits, and bovine species, most of the remaining ~ 5% of renal CA is membrane associated and consists of CA-IV and CA-XII [27] CA-II is expressed in the renal proximal tubule; thin descending limb; thick ascending limb; and intercalated cells of the cortical collecting duct, outer medullary collecting duct, and inner medullary collecting duct. Schwartz [28] described that the function of CA-II in renal H^+^/HCO_3_^-^ transport is perhaps best understood by examining CA-II interactions with specific transporters.

Räisänen et al., [29] suggested that CA-III is an oxyradical scavenger that protects cells from oxidative damage. Using HK-2 cells, which represent an established model for normal human proximal tubule cells, Gailly et al. [30] reported that exposure to 1 mM H_2_O_2_ induced a significant increase in CA-III mRNA expression. This suggests that CA-III may be a multifunctional enzyme, and that 1 of the functions is to protect cells from oxidative damage.

Recently, Pertovaara et al. [31] reported that the levels of anti-CA-VI antibody were significantly higher in patients with primary Sjogren’s syndrome (pSS). The amount of antibody correlated significantly with urinary pH, and inversely with serum sodium concentrations. Anti-CA-VI antibody seems to be associated with renal acidification capacity in patients with pSS. However, the role of CA-VI autoantibodies in modulating urinary pH in the kidney remained perplexing, since the presence of CA-VI has never been demonstrated in the human kidneys. Pertovaara et al. [31] speculated that anti-CA-VI antibodies might exhibit cross-reactivity with CA-XIII expressed in the kidneys. However, the molecular weight of CA-XIII is 30 kDa [2] and the subunit molecular weight of swine CA-VI is 37 kDa [8]. Furthermore, the amino acid sequence homology between human CA-VI and CA-XIII is only 35%, which is also the degree of homology between CA-VI and CA-II [2]. We feel it is unlikely that cross-reactivity explains the results above. In support of this, despite a 62% amino acid sequence homology between equine CA-I and CA-II, sera raised against each of these isoforms do not exhibit cross-reactivity with the other [32]. These results indicate that anti human CA-VI serum does not cross-react with both human CA-XIII and CA-II.

The exact function of CA-VI in the kidneys remains unclear at this stage. However, based on our preliminary data, additional studies should be conducted to determine whether measuring the CA-VI concentration in the urine of pigs with kidney disorders is of clinical utility.

## Conclusions

CA-VI was located in the epithelial cells of distal straight tubule of swine kidneys.

The cDNA sequence was 1348 base pairs long and was predicted to encode a 317 amino acid polypeptide with a putative signal peptide of 17 amino acids. The deduced amino acid sequence of mature CA-VI was most similar (77.4%) to that of human CA-VI. The expression of CA-VI was demonstrated in both normal and nephritic kidneys, and the relative levels of CA-VI mRNA in the nephritic kidneys were significantly lower than in normal kidneys. The level of CA-VI mRNA in normal kidneys was 19-fold lower than that of the parotid gland.

## Abbreviations

CA-VI: Carbonic anhydrase VI; RT-PCR: Reverse transcription- polymerase chain reaction.

## Competing interests

The authors declare that they have no competing interests.

## Authors’ contributions

TN, JY and HO were responsible for the study conception and design, development of the questionnaire, carried out the data collection, performed descriptive statistical analyses and drafted and revised the manuscript. KO helped in the descriptive statistical analyses. MM and KA helped in the design of the study and coordination. SK and NI performed the drafting of the manuscript. All authors read and approved the final manuscript.

## Acknowledgement

The authors declare that no funding support was obtained for this study.
